# Traumatic Spondylolisthesis of the Fourth Lumbar Vertebra Without Neurologic Deficit or Fracture of the Posterior Elements

**DOI:** 10.7759/cureus.13238

**Published:** 2021-02-09

**Authors:** Ioannis Papaioannou, Thomas Repantis, Georgia Pantazidou, Andreas Baikousis, Panagiotis Korovessis

**Affiliations:** 1 Orthopedics, General Hospital of Patras, Patras, GRC; 2 Orthopedics, Karamdaneion Hospital, Patras, GRC; 3 Otolaryngology - Head and Neck Surgery, General Hospital of Patras, Patras, GRC

**Keywords:** traumatic lumbar spondylolisthesis, pedicle screw fixation, ligamentous rupture, flexion/distraction injury

## Abstract

Acute traumatic spondylolisthesis in the lumbosacral spine is an uncommon injury. Traumatic dislocation of the fourth lumbar vertebra over the fifth lumbar vertebra (L4/L5) is extremely rare since few studies have been reported in the current literature. We report on a 53-year-old man, who had a motor vehicle accident and sustained an injury of the lumbar spine without neurological impairment. The radiographic evaluation disclosed an L4/L5 traumatic spondylolisthesis, classified as Meyerding grade III without any fracture of the posterior vertebral elements. To the best of our knowledge, this is the sixth case of L4 traumatic spondylolisthesis without concomitant fracture of the posterior vertebral elements and the third case without any neurological deficit among them. The patient underwent open reduction and posterior instrumentation. Intraoperatively, the posterior ligamentous complex, the capsules of the facet joints and also the disc were found torn, although facets, neural arch, and pedicles were intact. Following decompression and reduction of the spondylolisthesis without any neurologic complications, we performed pedicle screws and rods fixation from the third to the fifth lumbar vertebra (L3-L5). The patient had an uneventful recovery and returned to his previous activity three months after surgery. The four-year follow-up evaluation showed normal spinal alignment, successful pain-free fusion without neurologic complications. Flexion/distraction injury without simultaneous rotation at the L4/L5 segment during traffic accidents or the fall of a heavy object on the bent back accompanied with posterior ligament weakness is thought to be the probable mechanism for this type of injury. Concomitant neurologic impairment is associated with the majority of L4/L5 spondylolisthesis cases. Posterior decompression, reduction, and posterior instrumentation enhances bony fusion, improves the patient's neurologic status and restores the sagittal alignment.

## Introduction

Traumatic lumbar and lumbosacral spondylolisthesis is an uncommon injury. Especially, spondylolisthesis of the fourth lumbar vertebra (L4) is very rarely reported, while several cases with dislocation of the fifth lumbar vertebra (L5) over the first sacral vertebra (S1) have been described [1]. To our knowledge, there are only 16 studies with 18 patients reporting on L4 traumatic anterolisthesis, retrolisthesis, and spondyloptosis with or without concomitant fracture of the posterior vertebral elements [2-17]. Most of the patients in these reports were treated operatively with open reduction, intervertebral cages, pedicle screws, and rods fixation via the posterior approach. The purpose of this study is to report a rare case of L4 anterolisthesis without concomitant fracture of the posterior spinal elements, and also without neurologic impairment as well as to describe its operative treatment. We will also review all the published cases (according to our search on PubMed and Google Scholar) of L4 fracture-dislocation and pure dislocation, focusing on the mechanism of injuries, sagittal balance restoration, and neurological outcome following surgery. 

## Case presentation

**Figure 1:**
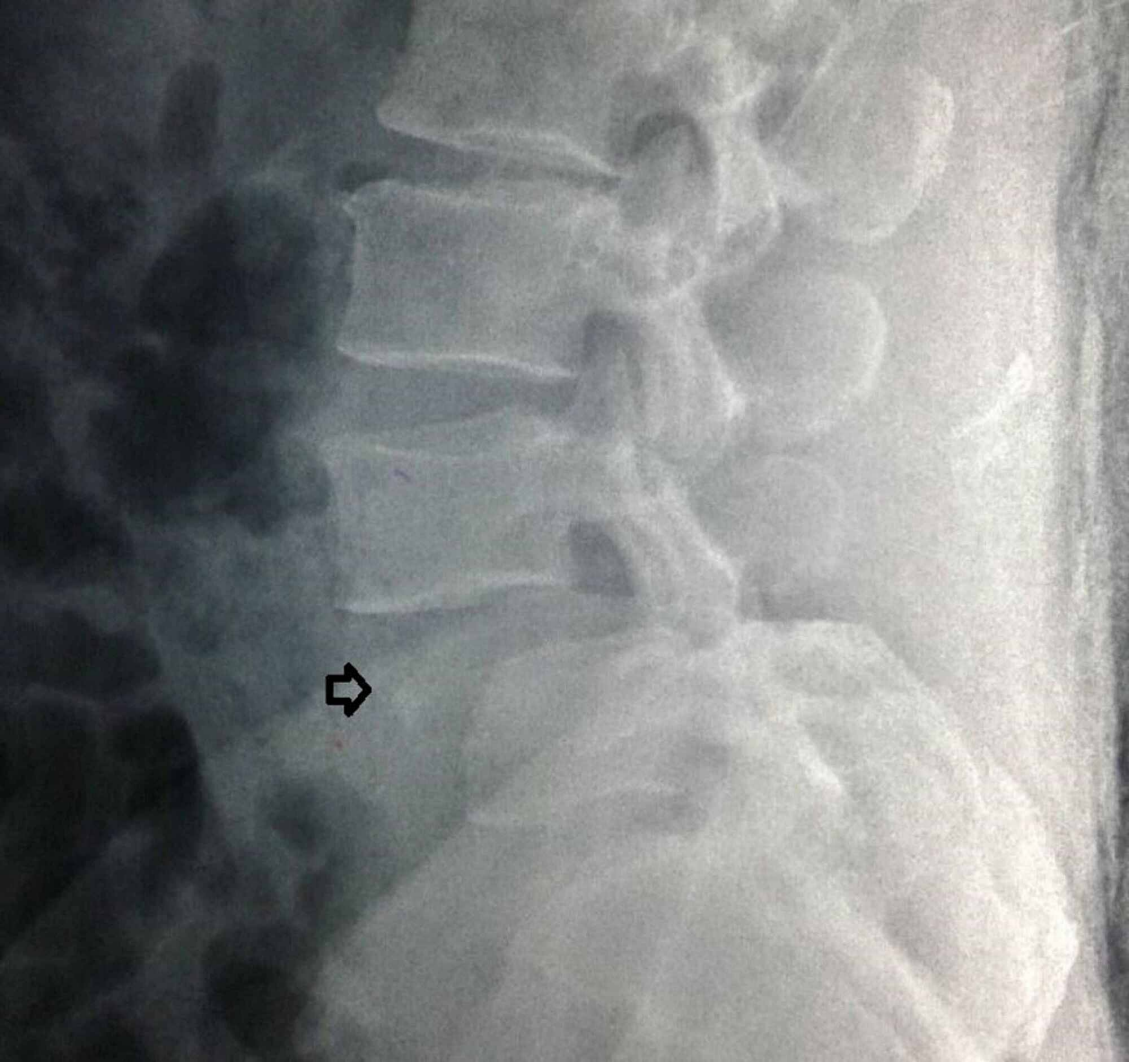
Preoperative supine lateral roentgenogram on admission of the lumbar spine showing Meyerding III anterolisthesis of L4. The black arrow shows the avulsion fracture of the anterior–superior vertebral corner.

**Figure 2:**
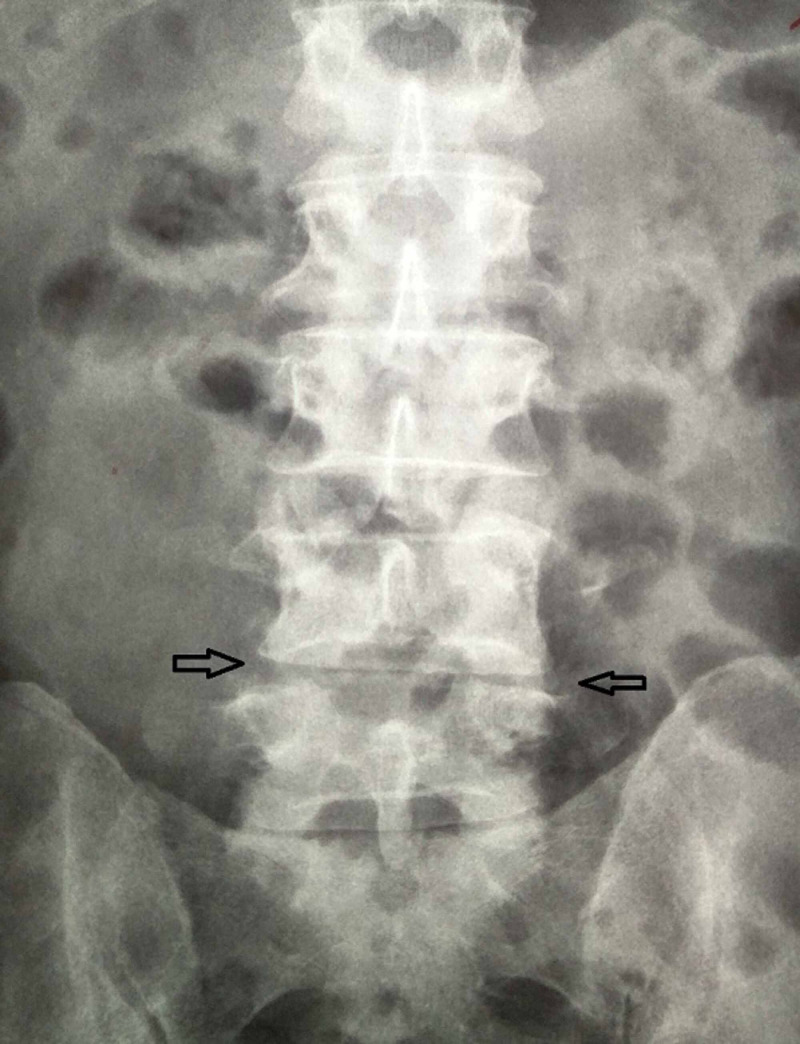
Preoperative anteroposterior roentgenogram of the lumbar spine showing narrowing of the intervertebral space (black arrows).

A 53-year-old man, with a body mass index (BMI) of 27.6 Kg/m2, was driving wearing his security belt with a velocity of about 70 Km/hour, when he lost control (with the same velocity) of his car and fell in a ditch from a height of about 1.5 meters. On admission, the patient was hemodynamically stable, while the physical examination disclosed swelling over the lower lumbar spine with severe pain on pressure without any neurologic impairment of the lower extremities. Plain roentgenograms in lying position revealed a traumatic dislocation of the fourth lumbar vertebra (L4), classified as Meyerding grade III spondylolisthesis (Figures 1, 2)

**Figure 3:**
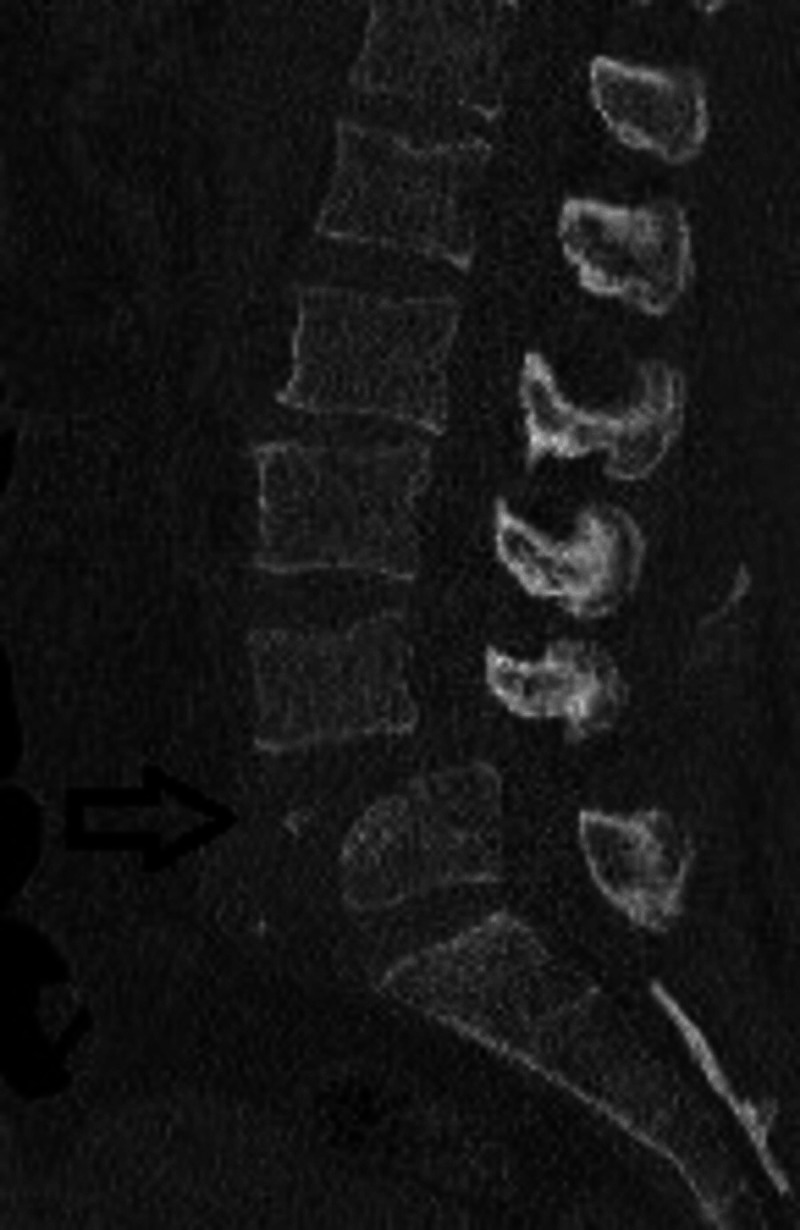
Preoperative lateral supine CT scan with sagittal reconstruction showing the anterolisthesis of L4 and the avulsion L5 fracture (black arrow).

**Figure 4:**
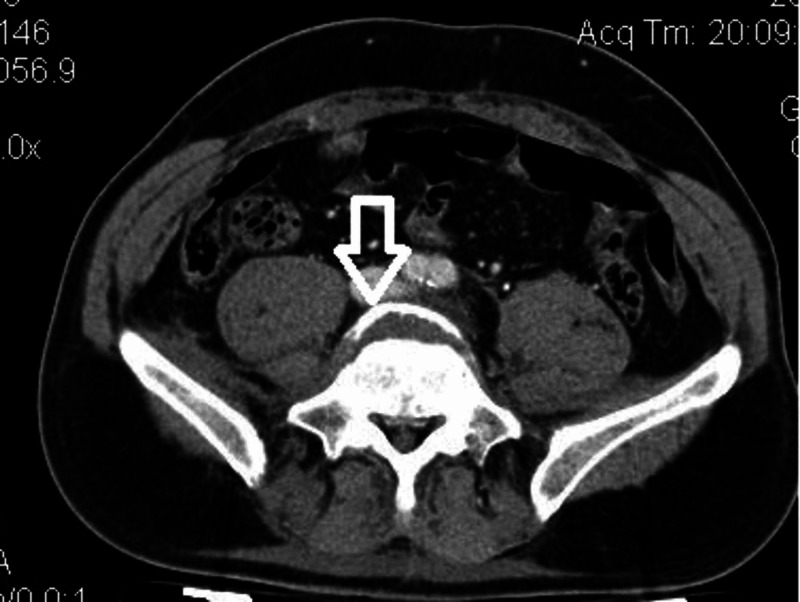
Preoperative axial CT scan at the segment L4-L5 showing double contour (white arrow), indication of olisthesis.

A full-body computed tomography confirmed the anterior dislocation of L4 vertebra associated with an avulsion fracture of the anterior/superior corner of the fifth lumbar vertebra (L5), accompanied by fractures of the transverse processes of the second, the third, and the fourth lumbar vertebrae, without, however, any fracture of the facet joints and neural arches (Figures 3, 4)

**Figure 5:**
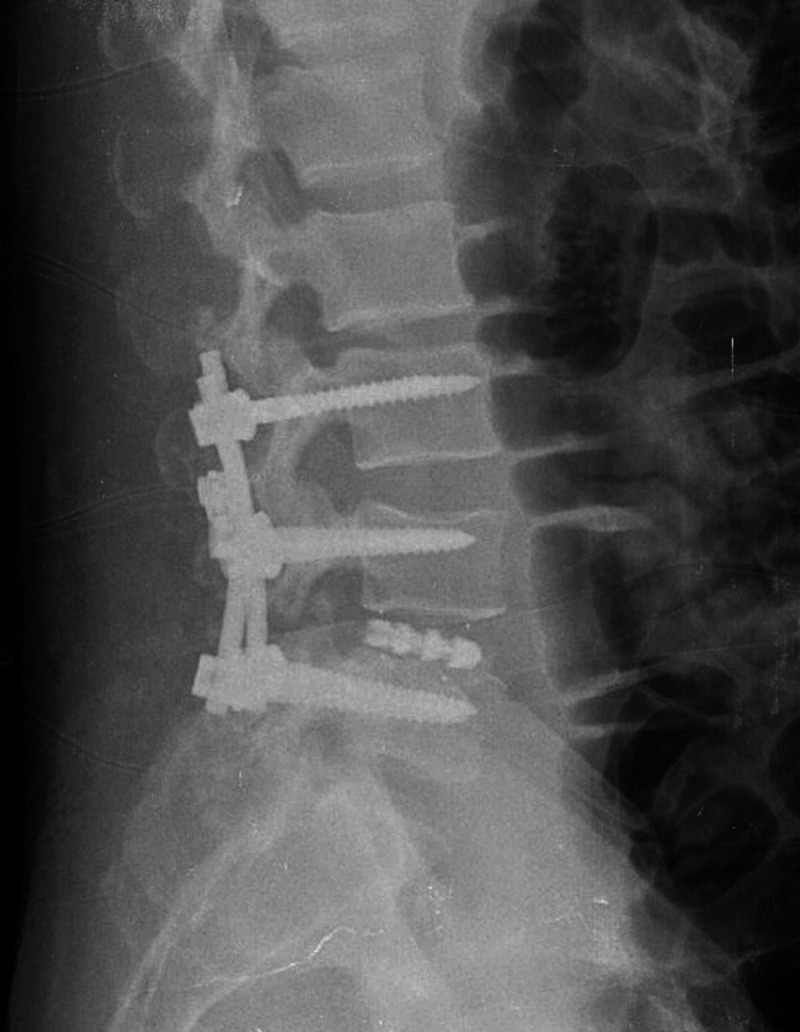
Postoperative (three months follow-up) standing roentgenogram of the lumbar spine showing excellent sagittal balance and pedicle screw-rod fixation in situ.

**Figure 6:**
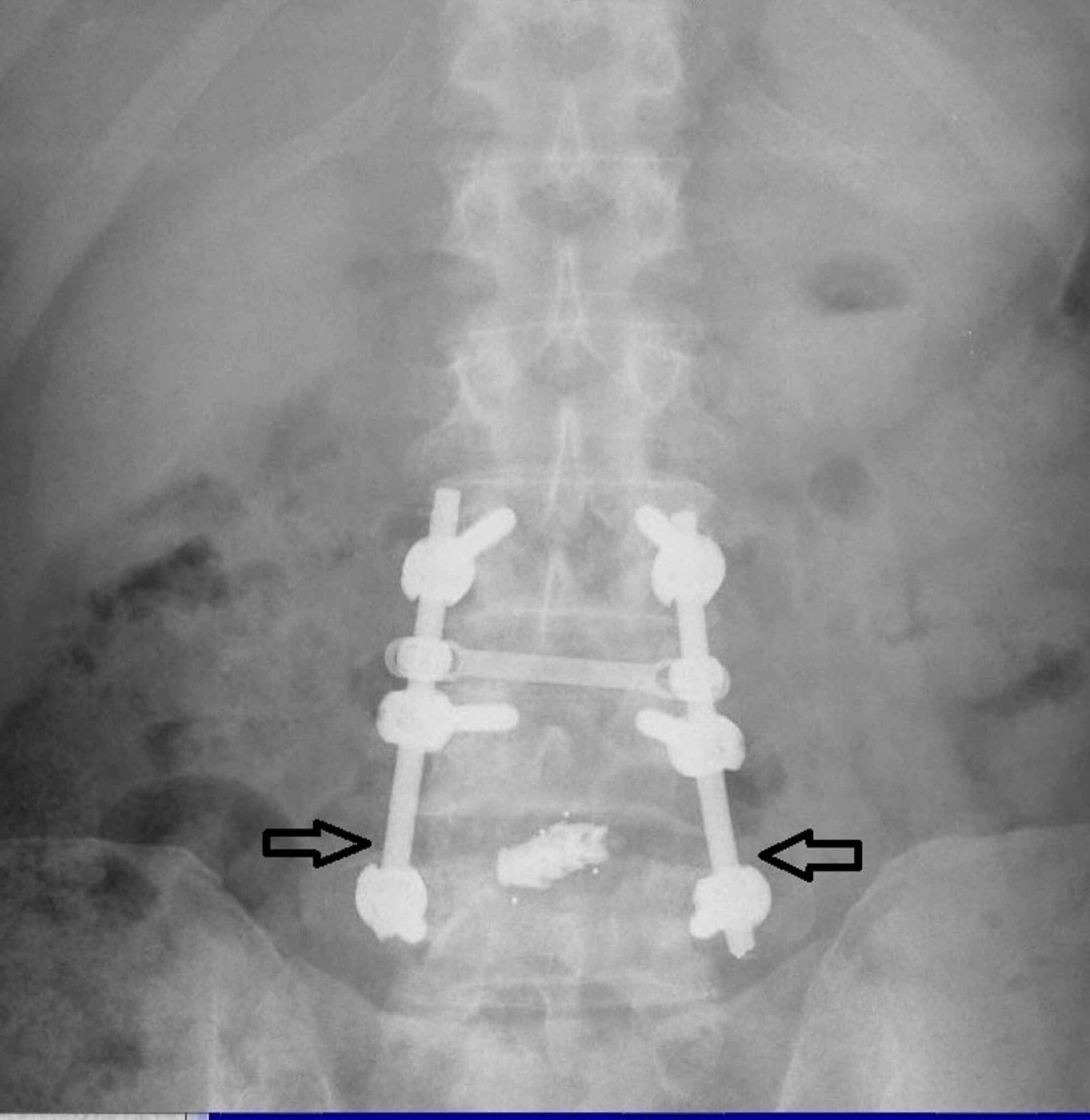
Postoperative (three months follow up) standing anteroposterior roentgenogram of the lumbar spine. The black arrows show the level of the interbody fusion.

The patient underwent open posterior decompression, reduction, and pedicle screw fixation within 24 hours following admission. Continuous neuromonitoring was used. During exposure, the posterior spinal ligamentous complex and facet joint capsules were found completely torn accompanied by bilateral anterior facet dislocation and rupture of the intervertebral disc, without any fracture of the facets or neural arches of L4 and L5 vertebrae. Decompressive laminectomy was made to facilitate the reduction of L4 vertebra and inspection of nerve roots and dural sac during the reduction maneuvers. Reduction of the dislocation was achieved using flexion/distraction maneuvers (leverage of the L4 vertebra using a laminar spreader, cobb elevators, and reduction pedicle screws through L4 pedicles) and it was temporarily secured with a unilateral longitudinal rod. An interbody expandable cage (transforaminal lumbar interbody fusion [TLIF]) was inserted in the L4/L5 intervertebral space after meticulous removal of the annulus fibrosus and the cartilage of the adjacent vertebral endplates. Autologous cortico-cancellous bone graft was impacted into the L4/L5 disc space. Finally, two lordotic contoured longitudinal rods were assembled with the screws and an autologous bone graft was placed posterolaterally to enhance fusion. The postoperative course was uneventful. The patient was mobilized on the first postoperative day with a brace and discharged on the third postoperative day. After a three-month follow-up, the patient was encouraged to progressively regain his previous activity as a pastry chef (Figures 5, 6)

**Figure 7:**
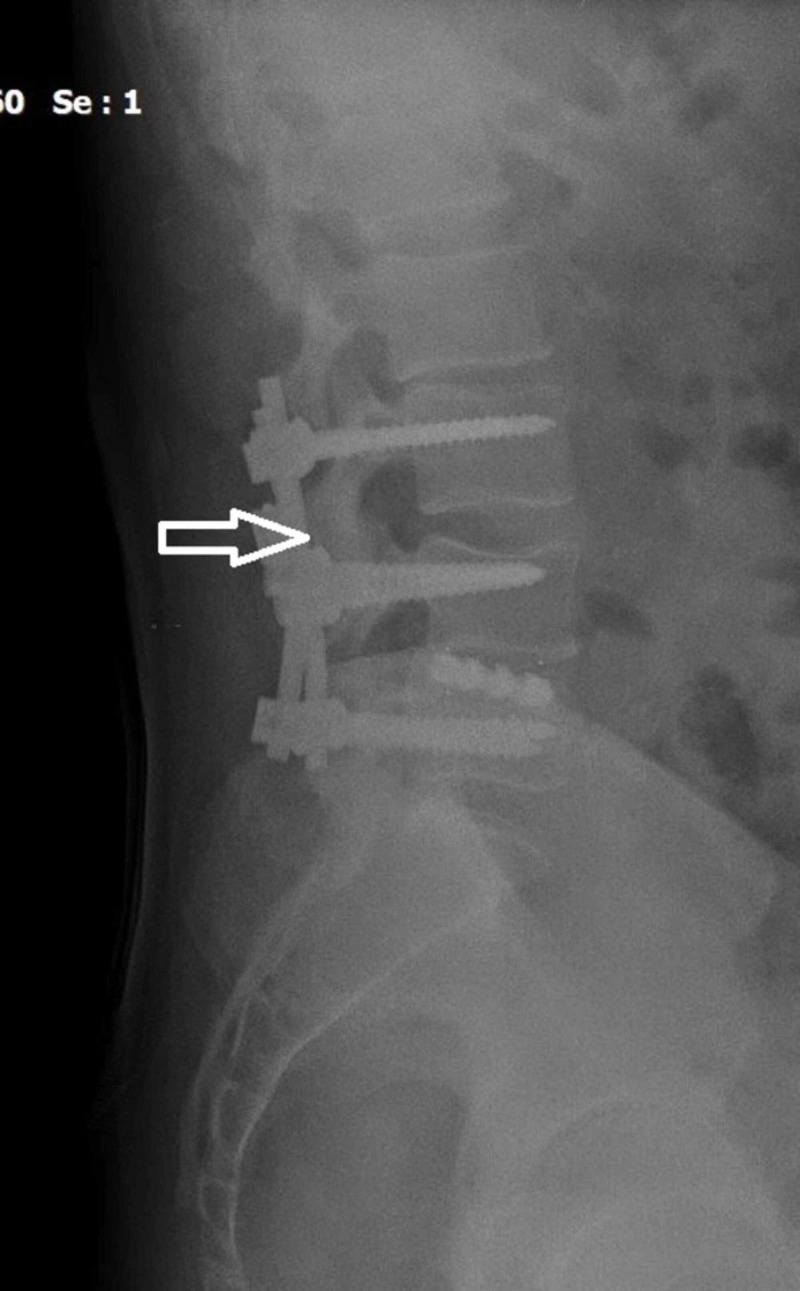
Postoperative lateral standing roentgenogram of the lumbar spine four years postoperatively. Note the completed fusion (white arrow) and excellent sagittal balance.

**Figure 8:**
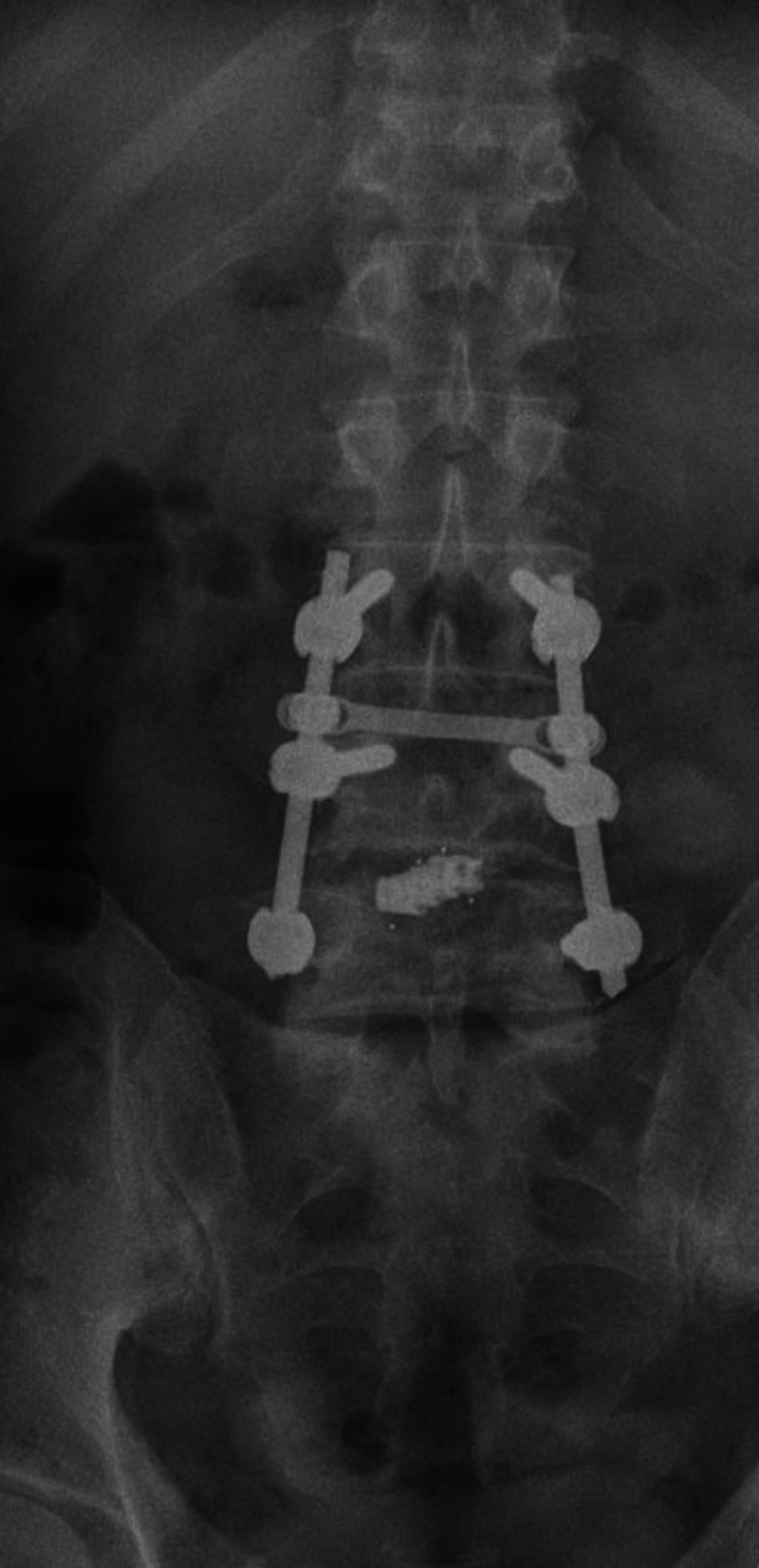
Postoperative anteroposterior standing lateral roentgenogram of the lumbar spine in the last evaluation, four years postoperatively.

At the latest follow-up, four years postoperatively, the patient was symptom-free with normal spinal alignment and complete posterolateral spinal fusion (Figures 7, 8)

## Discussion

Acute traumatic spondylolisthesis in the lumbosacral spine is not common. The combination of hyperextension, hyperflexion, and rotation has been described [1] as a pathogenic mechanism. Moreover, L4/L5 traumatic dislocation is extremely rare, since only 16 studies in the literature have reported anterolisthesis, retrolisthesis, or even spondyloptosis of L4, with or without neurologic impairment [2-17]. Among the 18 patients reported, only five cases have been reported with L4-anterolisthesis Meyerding III with “locked” but not fractured facets [5,15-17]. Concomitant neurologic impairment was reported in the majority of the cases (11 out of 18 cases) [3,4,9-13,15-17] (Table 1).

**Table 1:** Cumulative data on 16 published cases with traumatic olisthesis of L4. Case 2, 6a, 6b, 11, and 14 concern those without facet fracture, similar to our case.

No	Authors	Year	Cases	Gender	Age	Neurologic impairment on admission	Type of injury	Posterior vertebral elements fracture	Trauma to Surgery time	Type of surgery	Open/MIS	Follow up	Neurologic impairment on Follow up
1	Abdel-Fattah et al. [8]	1990	1	Female	18	ASIA E	fracture retrolisthesis L4, fall from 3 m height	fracture/dislocation retrolisthesis L4, all posterior elements fractured	Not available	sacral rod and two Harrington rods	Open primary & Revision surgey	7 months	ASIA E
2	Mori et al. [5]	2002	1	Female	32	ASIA E	Traumatic bilateral locked facet at L4-5 Meyerding III unusual seatbelt injury	pure dislocation associated with locked facet at L4-5	2 weeks	L4-L5 posterior pedicle screw plus PLIF	Open	18 months	ASIA E
3	Song et al. [6]	2005	1	Female	34	ASIA E	fracture dislocation L4, traffic	L5 facets fractures	3 months	Posterior L4-L5 plus PLIF	Open	12 months	ASIA E
4	Ahmed et al. [9]	2005	1	Female	34	Cauda Equina	traffic stucked fracture/dislocation retrolisthesis L4	L5 facets fractures	10 days	Posterior, decompression, L4–L5 PLIF titanium interbody cage, pedicle screws L3-L5	Open	24 months	ASIA E
5	Deniz et al. [2]	2008	1	Male	44	ASIA E	fracture dislocation L4, Meyerding I .Driver of a tractor that has crashed to a tree	L4 inferior facet fracture & bilateral facet dislocation	4 months	Posterior, decompression, L4–L5 interbody cage, pedicle screws L3-L5	Open	3 months	ASIA E
6a	Lim et al. [17]	2009	1	Male	41	Cauda Equina	a 600-kg container fell onto his back	fracture of the anterior-superior corner of L5 associated with a Chance-type fracture-dislocation through the L4/5 disc space and bilateral perched facets with grade 2 spondylolisthesis of L4 on L5	4 hours	Posterior decompression, L4–L5 pedicle screws and TLIF	Open	1 year	Almost full cauda function recovery
6b	Lim et al. [17]	2009	1	Male	56	ASIA E	falling from a height of 2 metres	comminuted fracture of L5 and grade-1 anterolithesis of L4 on L5	24 hours	Posterior decompression, L3-S1 pedicle screw fixation, L4-L5 TLIF	Open	1 Year	ASIA E
7a	Zhou et al. [3]	2010	1	Male	19	Cauda Equina	L4 spondyloptosis, heavy object fell on his back	L5 facets fractures	10 days	L4-L45 pedicle screws plus PLIF bone graft	Open	78 months	Almost full cauda function recovery
7b	Zhou et al. [3]	2010	1	Male	13	Cauda Equina	L4 spondyloptosis, heavy object fell on his back	facets and lamina of L5 fracture	5 days	L3-S1 pedicle screws	Open	12 months	Drop foot only
8	Chandrashekhara et al. [11]	2011	1	Male	10	Cauda Equina	spondyloptosis L4 over L5. He fell from a running truck	L3 and L4 facet fracture	Not available	Pedicle screws L2-L5	Open	Not mentioned	Mild improvement
9	Zarate-Kalfopulos et al. [10]	2012	1	Male	20	Cauda Equina	traffic accident dislocation of L4–L5. Influence of alcohol was in a car that rolled over	L4 and L5 facet fractures	3 days	Traction, then surgery L3-S1 pedicle screws	Open	12 months	ASIA D
10	Tang et al. [12]	2012	1	Female	48	incomplete cauda equina syndrom	Traffic stuch on a car	transverse process fractures of L2–3 on the left and L4 bilaterally, spinous process fractures of L2–4, and lamina fracture of L4. Compression of the common iliac artery by the L4 body and a triangular fracture fragment	13 hours	single stage combined anterior and posterior approach anterior PEEK cage	Open	24 months	ASIA D
11	Im et al. [15]	2012	1	Male	37	ASIA D	Anterior slippage of the 4th lumbar vertebra. Held down by an iron plate weighing 2,000 kg.	pure dislocation associated with locked facet at L4-5	Not available	L4-L5 posterior pedicle screw plus PLIF	Open	6 months	ASIA E
12	Amesiya et al. [4]	2014	1	Male	34	ASIA C	L4 spondyloptosis He was hit in the back	L4/5 level with disruption of the facet joints	4 days	L4-L5 pedicle screws	Open	2 months	ASIA D
13	Zenonos et al. [7]	2016	1	Male	36	ASIA E	Traffic	L4 fracture upper facet fracture	1 day	L3-L5 fusion pedicle screws	Open	3 months	ASIA E
14	N'Dri-Oka et al. [16]	2016	1	Male	33	incomplete cauda equina syndrom	road traffic accident	spondylolisthesis at L4-L5, without facet fracture	N/A	Decompression, L4-L5 pedicle screws plus TLIF	Open	16 months	ASIA D
15	Park et al. [14]	2018	1	Female	34	ASIA E	locked facet dislocation, traffic	L5 superior facet fractures	3 weeks	L4-L5 posterior pedicle screw plus PLIF	Open	12 months	ASIA E
16	Sasagawa [13]	2019	1	Male	60	ASIA D	Grade 3 traumatic spondylolisthesis of L4	Pedicle and lamina fracture L3 & L4	24 hours	2 stage anterior & posterior surgery/ 11 days interval plus PEEK via oblique lumbar interbody approach/ L2-S1	Open	24 months	ASIA D

To the best of our knowledge, our case is the sixth traumatic L4 anterolisthesis, Meyerding III reported, without associated fracture of the posterior vertebral elements (pedicles, facets, and neural arch), and the third case without neurological deficits caused by a trauma of moderate energy. According to the Denis classification, this is an unstable three-column injury, which requires surgical stabilization, as in all previously published cases. In our case, the probable mechanism for L4 olisthesis is a severe flexion-distraction injury in the lumbar spine with the safety belt acting as the fulcrum for the bending force applied to the spine. There was initially rupture of the posterior ligamentous complex (supraspinous, interspinous, ligamentum flavum, posterior longitudinal ligament, facet joint capsule, and intervertebral disc) and subsequently dislocation of the facet joints without fracture. All these were intraoperatively certified. Facet joints and their well-developed capsules play a crucial role in the stability of the lumbar spine [18]. Usually, a flexion-rotation injury is required to provoke dislocation of these structures [18]. In biomechanical studies, it has been reported that when vertebral displacement in the lumbar spine is observed, facet fracture is suspected, which permits abnormal axial rotation and subsequent dislocation [18]. However, in five previous cases [5,15-17] and in our case, no fracture was associated with the L4-L5 anterolisthesis. Among these five previously reported cases, only two patients were without neurologic deficits on admission [5,17]. So, our case is the third one which is associated with traumatic L4/L5 spondylolisthesis without fracture of the posterior elements and also without neurological impairment. Sullivan and Farfan [19] showed in a biomechanical study that axial rotation ≥30^o ^of the lumbar spine caused the failure of the neural arch, progressing from facet joint dislocation to fracture of the articular process. Thirteen out of 18 previously published cases with L4 spondylolisthesis were associated with facet fracture, while in our case and those of Mori et al. [5], Im et al. [15], N’Dri-Oka et al. [16], and Lim et al. [17], there was no facet fracture, but locking facets and pure dislocation. Apparently, the rupture of the facet joint capsule has occurred without simultaneous spinal rotation, resulting thus in a pure anterolisthesis. Aside from the mechanical forces that act in the lower lumbar spine, weakness of the posterior ligament complex has been suggested as a predisposing factor for lumbar fracture-dislocation in 21% of subjects aged >20 years [20]. Patients' age averaged 31.9±14.52 years in the published papers, but there were four patients with ages lower than 20 years [3,8,11] (Table 1). Eleven out of the 18 patients were admitted with neurologic impairment [3,4,9-13,15-17]. Neurologic injury was more common in patients with L4 retrolisthesis. However, all patients who had on admission neurological deficits showed worth noting improvement in the final observation (Table 1). Successful pedicle screw fixation was applied in all the patients, except in two cases with L4-spondylolisthesis [8,13]. The insertion of an intravertebral cage in 12 out of 18 cases [2,3,5,6,9,12-14,16,17] resulted in solid fusion and reportedly restoration of sagittal balance.

## Conclusions

In conclusion in our case, high-energy trauma resulted in disruption of the already weakened posterior ligamentous complex, anterior sliding of the L4 vertebra body, and a subsequent three-column injury, according to Denis classification. Open decompression, reduction with internal segmental pedicle screw fixation, and fusion is the most accepted treatment resulting in excellent short- and long-term clinical and radiological results and neurologic recovery of the patients. Clinicians and spine surgeons should be aware that traumatic lumbar spondylolisthesis can take place even with the absence of neurologic impairment and/or without any fracture of the posterior vertebral elements. 
